# Clavicle length and scapulothoracic angle in young children with internal rotation contracture secondary to brachial plexus birth palsy: a bilateral MRI study

**DOI:** 10.1016/j.xrrt.2026.100823

**Published:** 2026-07-17

**Authors:** Jourdan H. Meltzer, Michael L. Pearl

**Affiliations:** a Cedars-Sinai Medical Center (CSMC), Los Angeles, CA, USA; b Cedars-Sinai Medical Center (CSMC), Southern California Permanente Medical Group (SCPMG), Los Angeles, CA, USA

**Keywords:** Brachial plexus birth palsy, Clavicle length, Upper limb development, Pediatric orthopedics, Shoulder and elbow, Forequarter anatomy

## Abstract

**Background:**

The most common sequela of brachial plexus birth palsy (BPBP) is an internal rotation contracture (IRC) of the shoulder. The contracture is known to result primarily from restricted motion and deformity at the glenohumeral joint, and, to a lesser extent, decreased humeral retroversion (HRV). Limb length discrepancies of the affected arm are also common, raising the question of whether a shortened clavicle alters the orientation of the scapula into more internal rotation to further limit external rotation.

**Hypothesis:**

We hypothesized that on the affected side of young children with IRCs from BPBP, the clavicle length (CL) and scapulothoracic angle (STA) would be reduced, and their reduction would correlate with each other and the severity of the IRC.

**Methods:**

In this retrospective study, 24 children with a median age of 1.5 years (range: 0.5 to 10.1), as part of treatment for shoulder IRC secondary to BPBP, underwent magnetic resonance imaging inclusive of both humeri, scapulae, clavicles, and thorax. Measures of bilateral STA, CL, glenoid retroversion, and HRV were made. Interobserver and intraobserver reliability were assessed. Paired *t*-tests were performed to compare STA, CL, glenoid retroversion, and HRV between involved and uninvolved sides, with *P* value<.05 considered statistically significant. Spearman correlation coefficients were applied to assess associations between age (at magnetic resonance imaging), IRC severity, ratio of CL (involved/uninvolved), and difference in STA.

**Results:**

On the affected side, CL was significantly reduced (67.2 mm, range: 60.2-124.7, vs. 79.2 mm, range: 57.2-129.0; *P* < .001), corresponding to 94.5% of the uninvolved side (range: 70.5%-107.1%). STA was also significantly reduced on the involved side (37.8°, range: 27°-53°, vs. 42.7°, range: 27.0°-53.0°; *P* = .001), with a mean difference of 5°. In this cohort, CL and STA did not correlate with each other or with the severity of the IRC.

**Conclusions:**

Young children with IRC from BPBP sequelae commonly have reduced CL and an altered STA on the involved side. In this skeletally immature cohort, these differences were not correlated with contracture severity, suggesting that they represent only one aspect of the overall pathoanatomy. Quantitively, these findings constitute a small angular component of the contracture severity but are useful in understanding the appearance of the shoulder girdle and counseling families of young children. A study of older children (after the secondary growth spurt) is warranted to assess whether these changes progress further, accentuating upper limb deformity and limiting motion.

The most common sequela of brachial plexus birth palsy (BPBP) is an internal rotation contracture (IRC) of the shoulder.^9^^,^^11^^,^^20^ The causes of this contracture are multifactorial, beginning with external rotation (ER) weakness, followed by capsular contracture, and likely diminished growth of the subscapularis.^20^ The consequent dysplasia of the glenohumeral joint is notable for increased glenoid retroversion (GRV) with formation of a pseudoglenoid with posterior displacement of a flattened humeral head relative to the scapula.^4^,^12^^,^^13^ More recent research has demonstrated that decreased retroversion of the humeral shaft commonly occurs and further contributes to the internal posture of the arm.^10^^,^^16^^,^^18^

There is less understanding regarding the consequences of brachial plexus injury on the development of the proximal part of the forequarter, specifically the clavicle and the orientation of the scapula to the thorax. Asymmetrical scapular posture and motion are clinically well known in this condition and even form the basis of one of the oldest reported findings associated with IRCs, the Putti sign.^3^ Attempts to quantify changes to scapular position have been challenged by its complex shape, position, and motion.^8^^,^^15^ The clinical measure of ER, by convention, is the angular measure of the forearm relative to the thorax. Altered shape and/or alignment of any of the multiple skeletal segments inclusive of and between the arm and thorax may alter the range of shoulder ER and contribute to an IRC.^13^^,^^14^

Clinically, a foreshortened clavicle is often apparent in these patients but has not been the focus of clinical evaluation or treatment thus far. A recent study by Rosenauer et al^17^ did find differences in clavicle length (CL) and altered scapular position in an older cohort with BPBP sequela but did not evaluate or report on the presence of an IRC. The purpose of our study is to specifically assess children with an IRC of the shoulder to better understand how clavicular length and the resting posture of the scapula on the thorax relate and contribute to the IRC. The hypotheses tested were that, in a cohort of children receiving treatment for their IRC, there are side-to-side differences in clavicular length and the resting posture of the scapula on the thorax between their involved and uninvolved sides that correlate with the severity of their IRC and each other.

## Material and methods

In this retrospective study, 24 children with a median age of 1.5 years (range: 0.5 to 10.1), as part of treatment for shoulder IRC secondary to BPBP, underwent magnetic resonance imaging (MRI) inclusive of both humeri, scapulae, clavicles, and thorax were included for analysis (Table I). In our practice, passive ER with the arm at the side that is 0° or less, or 45° less than the unaffected side, is considered an IRC and is indicated for treatment. All children except 1 went on to further intervention, 2 with Botox only, the remainder with a combination of nerve transfers (4), shoulder releases (15), latissimus transfers (8), and humeral osteotomies (4). For the purpose of this study, passive ER was measured under general anesthesia at the time of Botox or surgical intervention, except for one child who did not undergo such intervention and was assessed in clinic. IRC severity was quantified as a decrease in passive ER. See Supplementary Figs. 1 and 2 for methodology of MRI measurement.

**Table I:** Demographics of study population detailing the patient age at the time MRI was obtained, sex (F = female, M = male), side involved, glenoid type, and passive external rotation (measured under anesthesia in all but one patient).

Age at MRI (years old)	Sex	Side involved	Glenoid type	Passive external rotation (degrees)
0.5	F	Left	Pseudoglenoid	−30
2.8	F	Left	ConcentricPosterior	−10
0.5	F	Right	Pseudoglenoid	0
0.7	F	Right	Pseudoglenoid	20
1.8	M	Right	ConcentricPosterior	10
1.5	F	Right	Pseudoglenoid	20
10.0	F	Right	ConcentricPosterior	−30
4.2	M	Right	Pseudoglenoid	−30
0.8	F	Right	Pseudoglenoid	−20
9.9	M	Right	Pseudoglenoid	−20
1.5	F	Left	ConcentricPosterior	20
2.7	F	Right	ConcentricPosterior	0
0.6	F	Left	Pseudoglenoid	0
0.5	F	Right	Pseudoglenoid	−60
1.4	F	Right	Pseudoglenoid	10
0.9	F	Left	Concentric	0
10.1	F	Left	Pseudoglenoid	−30
0.9	F	Right	Biconcave	0
0.5	M	Right	Pseudoglenoid	−20
4.0	M	Left	Pseudogleniod	−60
3.5	F	Right	ConcentricPosterior	10
0.8	F	Left	Pseudoglenoid	−30
6.9	F	Right	Concentric	0

*MRI*, magnetic resonance imaging.

All analyses were 2-sided and performed using SAS EG 8.2 (Cary, NC). *P* values of less than 0.05 were considered statistically significant. Paired *t*-tests were performed to compare CL, scapulothoracic angle (STA), GRV, and humeral retroversion (HRV) between uninvolved and involved sides. Spearman correlation coefficients were applied to assess the associations between age (at MRI), severity of IRC (ER), ratio of CL (involved/uninvolved), and difference in STA.

Data were summarized with descriptive statistics as the mean (with standard deviation) and median (interquartile range) for continuous variables and as number (percentage) for categorical variables. Interobserver and intraobserver reliability for CL and STA measurements were assessed using 2-way random-effects models with absolute agreement. Reliability coefficients are reported in the Results section.

## Results

There was a significant reduction in the length of the clavicle of the affected side. The median length of the involved clavicle was measured at 67.2 mm (range: 60.2 to 124.7) vs. 79.2 mm (range: 57.2 to 129.0) on the uninvolved side for a *P* = .00048. Expressed as a percentage, the involved CL was 94.5% (range: 70.5% to 107.1%) as long as the uninvolved side. The median STA on the involved side was 37.8° (range: 27° to 53°) vs. 42.7° (range: 27.0° to 53.0°) on the uninvolved side.

Interobserver and intraobserver reliability were high. Interobserver ICCs were 0.90 for involved side CL, 0.91 for uninvolved side CL, 0.79 for involved side STA, and 0.73 for uninvolved side STA. Intraobserver reliability ranged from 0.76 to 0.94.

Spearman correlation coefficients did not reveal strong relationships between STA or CL and other clinical or imaging parameters, including the severity of IRC, age, or glenoid deformity (Table II). IRC severity was correlated with decreased GRV (*P* = .040) and decreased HRV (*P* = .035). However, no statistically significant correlation was observed between age and any of the measured anatomic variables: CL (*P* = .1), STA (*P* = .2), GRV (*P* = .9), or HRV (*P* = .3).

**Table II:** Results of multivariate regression with Spearman correlation coefficients for assessing associations between age, external rotation, clavicle length, scapulothoracic angle, glenoid retroversion, and humeral retroversion.

Independent variable	Age	ER	CL	STA	GRV	HRV
Age	1	−0.11118	−0.31813	0.26473	0.02924	0.20472
		0.605	0.1298	0.2112	0.8921	0.3373
ER	−0.11118	1	−0.30752	0.1224	0.42124	−0.18451
	0.605		0.1438	0.5688	0.0404	0.3881
CL	−0.31813	−0.30752	1	−0.13177	−0.11913	0.36486
	0.1298	0.1438		0.5394	0.5793	0.0796
STA	0.26473	0.1224	−0.13177	1	0.12698	−0.15724
	0.2112	0.5688	0.5394		0.5543	0.4631
GRV	0.02924	0.42124	−0.11913	0.12698	1	0.12655
	0.8921	0.0404	0.5793	0.5543		0.5557
HRV	0.20472	−0.18451	0.36486	−0.15724	0.12655	1
	0.3373	0.3881	0.0796	0.4631	0.5557	

*ER*, external rotation; *CL*, clavicle length; *STA*, scapulothoracic angle; *GRV*, glenoid retroversion; *HRV*, humeral retroversion.

## Discussion

In this cohort of young children with IRC secondary to sequelae of BPBP, the involved side demonstrated significantly shorter clavicular length and a reduced STA as compared with the uninvolved side. Although neither measurement correlated with the other, nor IRC severity, there are important potential clinical implications. For one, it is important to emphasize that all the children in this study were under 10 years of age, presumably prior to their secondary growth spurt. The other related study on this subject by Rosenauer et al^17^, additionally studied older children, some approaching or at skeletal maturity (oldest patient 23 years old), did show these 2 measures correlated. Unfortunately, their study did not measure ER of the shoulder or evaluate for the presence of an IRC, so the question as to whether these changes correlate with contracture severity in older children remains unanswered. Although not formally studied and not part of the present report, our clinical experience with older children with BPBP sequelae has shown CL and scapular posture pose significant concern for patients and their families with cosmetic and functional consequences. We have found a role for clavicle lengthening in select older patients after skeletal maturity presenting with cosmetic and functional consequences.^22^

Our finding of reduced CL on the involved side is consistent with a prior study of other segments of the upper extremity showing growth inhibition as a sequela of BPBP. Assessing side-to-side disparities in upper limb lengths following BPBP, Matsumura et al published on 22 children with radiographic markers, documented that the involved humerus and involved forearm were on average 93% and 90% the length of the uninvolved side.^5^ In contrast to our study with mostly upper plexus injuries, over half of their patients had lower plexus involvement, and consequently forearm lengths were more severely affected, 84% vs. 96% for upper trunk injuries.^6^ In another study of 48 children with upper and lower plexus injuries, average age 3.9 years, Bae et al^1^ found the affected limb segments similarly reduced to 95% for the upper arm, 94% for the forearm, and 97% for the hand. Terzis et al published on scanograms of 54 children, mean age 2.2 years, who were severely affected with 78% pan plexus injuries.^19^ Humeral and ulnar lengths were 95% and 93.2% on the involved side.^15^ van Gelein Vitringa et al reported the scapular width of 58 infants/toddlers (mean age 20 months) with plexus injuries showing a reduction of width on the involved sides of 90% or greater.^21^

Concluding from these prior studies and our study, BPBP will result in a smaller upper extremity that is on average 95% the length of the uninvolved limb, with more severe injuries lowering this percentage (71% lowest in the present study) and involvement of the lower plexus affecting the forearm and hand to a much greater extent.^1^^,^^2^ Age has not been found to correlate with the proportion of the size discrepancy. And finally, our study and the contemporaneous study by Rosenauer et al found similar growth differences in the clavicle.^17^

This study had several limitations. First, we had a small sample size due to the rarity of this condition and the complexity of obtaining MRIs in young children with requirement for anesthesia. CT mitigates some of this challenge but has the issues discussed above, namely, to reiterate, radiation exposure in young patients and inability to clearly delineate the cartilaginous structures of study. Another potential limitation of ours and other studies is that our angular measurements were made with the patient supine altering scapular position. Upright imaging with CT is possible, but it is impractical in this age group as most are too young to stand and cooperate for an upright study. Additionally, Matsumura et al^5^ studied the three-dimensional difference in alignment of the shoulder between upright and supine computed tomography scans and found only a 2° difference in scapular internal rotation between positions, with that in the supine position being greater than that standing. Furthermore, others faced with the reality of imaging this patient population have made similar measurements with patients in the supine position.^7^^,^^8^^,^^17^

Lastly, reducing the 3-dimensional S-shaped complexity of the clavicle bone to a single linear measure of length and the 3D orientation of the scapula on the thorax to a single angle must also be considered a limitation, as it clearly underrepresents the complex changes that affect the forequarter anatomy of these patients. Melillo et al^7^ studied a range of clavicle features in normal adults (length, curvature, etc.) and how they influence the position of the scapula on the thorax, finding that orientation on the thorax is equally important. The complexity of this bone alone merits further study in the brachial plexus population as well.

## Conclusions

Young children with an IRC secondary to BPBP, CL, and STA of the affected shoulder are commonly decreased as compared with their unaffected side. These findings allow for more informed counseling and management of patients and their families. The altered appearance of the shoulder can be explained in part by the shortened clavicle and mal-rotated scapula, which may in small measure contribute to the IRC. Clinically, these findings do not support clavicle lengthening as a means of improving ER in skeletally immature children. In an older child or adult with pronounced asymmetry, however, the role of surgical intervention merits further study and discussion.

## Disclaimers:

Funding: No funding was disclosed by the authors.

Conflicts of interest: The authors, their immediate families, and any research foundations with which they are affiliated have not received any financial payments or other benefits from any commercial entity related to the subject of this article.
