# Supplementary material for: Clavicle length and scapulothoracic angle in young children with internal rotation contracture secondary to brachial plexus birth palsy: a bilateral MRI study
Source: JSES Rev Rep Tech. 2026 Jul 17;6(4):100823. doi: 10.1016/j.xrrt.2026.100823 (PMC13499186; doi:10.1016/j.xrrt.2026.100823)
Supplement: Supplementary file 1 [file mmc1.docx]

Figure 1: Axial MRI cut showing the measure of the scapulothoracic angle (STA) on the involved (inv) and uninvolved (Uninv) sides. STA was measured as the angle formed by the intersection a midline (drawn from center of sternum through center of vertebral body) and a scapular line (midline along the long axis of the scapular body).

Figure 1: Axial MRI cut showing the measure of the scapulothoracic angle (STA) on the involved (inv) and uninvolved (Uninv) sides. STA was measured as the angle formed by the intersection a midline (drawn from center of sternum through center of vertebral body) and a scapular line (midline along the long axis of the scapular body).
