# Supplementary material for: Clavicle length and scapulothoracic angle in young children with internal rotation contracture secondary to brachial plexus birth palsy: a bilateral MRI study
Source: JSES Rev Rep Tech. 2026 Jul 17;6(4):100823. doi: 10.1016/j.xrrt.2026.100823 (PMC13499186; doi:10.1016/j.xrrt.2026.100823)
Supplement: Supplementary file 2 [file mmc2.docx]

Sternoclavicular\	.,---... (SC) Joint	• �

Acromioclavicular (AC) Joint

**Proximal	Distal**

Figure 2: Illustration of clavicle length measurement and corresponding coronal MRI cuts identifying coordinates of the proximal and distal ends. Clavicle length was calculated using three-dimensional coordinates designating the proximal (sternoclavicular joint) and distal ends (acromial clavicular joint) of the clavicle from MRI studies of bilateral shoulders.
